# Machine Learning–Based Analysis of Lifestyle Risk Factors for Atherosclerotic Cardiovascular Disease: Retrospective Case-Control Study

**DOI:** 10.2196/74415

**Published:** 2025-08-07

**Authors:** Hye-Jin Kim, Heeji Choi, Hyo-Jung Ahn, Seung-Ho Shin, Chulho Kim, Sang-Hwa Lee, Jong-Hee Sohn, Jae-Jun Lee

**Affiliations:** 1 Artificial Intelligence Research Center, College of Medicine, Hallym University, Chuhcneon-si, Republic of Korea; 2 Health Insurance Review and Assessment Research Institute, Health Insurance Review and Assessment Service, Wonju-si, Republic of Korea; 3 Department of Neurology, College of Medicine, Hallym University, 1, Hallymdaehak-gil, Chuncheon-si, 24252, Republic of Korea, 82 332405255, 82 1095173201; 4 Department of Anesthesiology, College of Medicine, Hallym University, Chuncheon-si, Republic of Korea

**Keywords:** atherosclerotic cardiovascular disease, machine learning, lifestyle risk behaviors, predictive modeling, low-density lipoprotein

## Abstract

**Background:**

The risk of developing atherosclerotic cardiovascular disease (ASCVD) varies among individuals and is related to a variety of lifestyle factors in addition to the presence of chronic diseases.

**Objective:**

We aimed to assess the predictive accuracy of machine learning (ML) models incorporating lifestyle risk behaviors for ASCVD risk using the Korean nationwide database.

**Methods:**

Using data from the Korea National Health and Nutrition Examination Survey, 5 ML algorithms were used for the prediction of high ASCVD risk: logistic regression (LR), support vector machine, random forest, extreme gradient boosting, and light gradient boosting models. ASCVD risk was assessed using the pooled cohort equations, with a high-risk threshold of ≥7.5% over 10 years. Among the 8573 participants aged 40‐79 years, propensity score matching (PSM) was used to adjust for demographic confounders. We divided the dataset into a training and a test dataset in an 8:2 ratio. We also used bootstrapping to train the ML model with the area under the receiver operating characteristics curve score. Shapley additive explanations were used to identify the models’ important variables in assessing high ASCVD risks. In sensitivity analysis, we additionally performed binary LR analysis, in which the ML model’s results were consistent with the conventional statistical model.

**Results:**

Of the 8573 participants, 41.7% (n=3578) had high ASCVD risk. Before PSM, age and sex differed significantly between groups. PSM (1:1) yielded 1976 patients with balanced demographics. After PSM, the high ASCVD risk group had higher alcohol or tobacco use, lower omega-3 intake, higher BMI, less physical activity, and spent less time sitting. In 5 ML models, the extreme gradient boosting model showed the highest area under the receiver operating characteristics curve, indicating superior overall discrimination between high and low ASCVD risk groups. However, the light gradient boosting model demonstrated better performance in accuracy, recall, and *F*_1_-score. Variable importance analysis using Shapley additive explanations identified smoking and age as the strongest predictors, while BMI, sodium or omega-3 intake, and low-density lipoprotein cholesterol also had significant variables. Sensitivity analysis using multivariable LR analysis also confirmed these findings, showing that smoking, BMI, and low-density lipoprotein cholesterol increased ASCVD risk, whereas omega-3 intake and physical activity were associated with lower risk.

**Conclusions:**

Analyzing lifestyle behavioral factors in ASCVD risk with an ML model improves the predictive performance compared to traditional models. Personalized prevention strategies tailored to an individual’s lifestyle can effectively reduce ASCVD risk.

## Introduction

Atherosclerotic cardiovascular disease (ASCVD) remains the leading cause of morbidity and mortality worldwide [1]. In 2019, cardiovascular diseases (CVDs) were responsible for an estimated 32% of all global deaths [2]. Of the 17 million premature deaths caused by noncommunicable diseases in 2019, 38% were attributable to CVDs [2]. The prevalence of CVDs in South Korea has recently been increasing [3]. While the CVD mortality rate declined until 2010, it has been steadily rising since then [4]. Considering these trends, addressing CVDs is crucial for improving public health outcomes.

The main risk factors for developing ASCVD include hypertension, smoking, and dyslipidemia [5]. Additionally, ASCVD outcomes vary across different ethnic groups based on socioeconomic status, which also influences health-related lifestyle behaviors [6-8]. As the prevalence of ASCVD is increasing, the importance of primary prevention has been emphasized from a public health perspective [9]. ASCVDs are closely related to modifiable risk factors that can be controlled through lifestyle modification, which is the cornerstone of ASCVD prevention [9]. The most effective prevention strategy is a comprehensive approach that promotes a healthy lifestyle and addresses all major risk factors [10].

Machine learning (ML) has been increasingly applied in medical data analysis, offering numerous benefits for enhancing patient care and improving clinical outcomes [11]. Additionally, ML facilitates precision medicine by enabling early identification of high-risk patients for disease progression with high accuracy [11]. Previous studies have constructed ML models for predicting ASCVD, but few have modeled the impact of lifestyle behaviors on ASCVD risk [11-13]. Despite advances in ML, the combined predictive power of detailed lifestyle factors—especially within interpretable ML frameworks—remains underexplored. Although ML improves risk prediction, understanding which lifestyle factors have the greatest impact is crucial for prevention—yet this is often unclear with black-box models. This study addresses that gap by applying interpretable ML techniques to accurately predict ASCVD risk and clarify the specific impact of individual lifestyle factors. ASCVD risk is influenced by demographic variables such as age, race, and sex, as well as lifestyle behaviors [14,15]. Using data from the nationwide Korea National Health and Nutrition Examination Survey (KNHANES), we used five ML models to (1) examine the relationship between lifestyle factors and ASCVD risk, (2) evaluate the predictive performance of these models, and (3) identify the most influential predictors using interpretable methods.

## Methods

### Participants

This retrospective case-control study used nationally representative data from the KNHANES. The KNHANES is an ongoing, cross-sectional survey initiated in 1998 by the Korea Disease Control and Prevention Agency (KDCA) to assess the health and nutritional status of the Korean population. We used anonymized, publicly available data from 2019 to 2021. Among the 22,559 total participants in the 2019‐2021 KNHANES dataset, we initially screened 10,481 individuals aged 40‐79 years with no history of cardiovascular events. We excluded 1908 participants with missing data on sodium intake, low-density lipoprotein (LDL) cholesterol, time spent sitting, occupation, marital status, smoking status, income, BMI, or weight change over 1 year. The final analytic sample included 8573 participants.

### Ethical Considerations

The original KNHANES surveys received ethical approval from the KDCA Institutional Review Board, and informed consent was obtained from all participants. All data were anonymized and deidentified by KDCA prior to public release. As this study involved the secondary analysis of deidentified public data, it was exempt from additional institutional review board review by our institution (2024-03-008).

### Measurement of ASCVD Risk Score

The 10-year ASCVD risk was assessed using the American College of Cardiology/American Heart Association pooled cohort equations (PCEs) risk score [15]. They recommended that PCEs for non-Hispanic White people may be considered for risk estimation in populations other than non-Hispanic African American and non-Hispanic White people [15,16]. Therefore, the estimation function of the ASCVD risk score for the White population was used in this study. This score algorithm is designed to predict both the 10-year (40‐79 years of age) and lifetime (20‐59 years of age) risk of ASCVD. This includes the assessment of both fatal and nonfatal coronary heart disease and stroke [17,18]. We categorized participants into two groups based on their 10-year ASCVD risk calculated using the PCEs: a low ASCVD risk group (ASCVD risk <7.5%) and a high ASCVD risk group (ASCVD risk ≥7.5%).

### Measurement of Lifestyle Risk Behaviors

The lifestyle risk behaviors analyzed in this study included sex, age, occupation, marital status, residential area, household income, alcohol consumption, smoking, omega-3 intake, sodium intake, BMI, weight change over 1 year, LDL cholesterol, physical activity, and time spent sitting. The occupation was classified into three categories of nonmanual workers, manual workers, and other workers. Marital status was separated and widowed, and divorced participants were assigned the no spouse status. The residential area was grouped as urban or rural areas. Alcohol consumption was divided into two categories, where participants drank at least 2 days per week and fewer than 2 days per week [19], regardless of the amount of alcohol consumed. Individuals who had smoked at least 100 cigarettes in their lifetime and currently smoked were classified as smokers, whereas those who had smoked fewer than 100 cigarettes in their lifetime but did not currently smoke and never smoked in their lifetime were classified as nonsmokers. BMI was calculated as weight in kilograms divided by height in meters squared. Weight change over 1 year was classified into three categories of no weight change, weight gain, and weight loss. Using the criteria specified in the questionnaire, weight gains and losses of 0 to less than 3 kg over 1 year were classified as no change, gains of more than 3 kg as weight gain, and losses of more than 3 kg as weight loss. Physical activity was calculated using the sum of the minutes of exercise per week and considering the strength of the exercise intensity (vigorous and moderate). The combination of vigorous intensity and moderate intensity exercise was considered by calculating the minutes exercised per week as follows: 2×moderate activity (minutes per week)+vigorous activity (minutes per week) [20]. “Physically active” is defined as engaging in at least 150 minutes of moderate activity per week [21]. We used omega-3 or sodium intake in grams per day and time spent sitting in minutes per day, and these variables were entered into the ML model as continuous variables.

### Statistical Analysis

We categorized ASCVD risk into two groups: high and low risk. The ASCVD risk score itself is calculated using age and sex. Consequently, the high-risk and low-risk groups are inherently expected to differ significantly in these demographic factors by definition. In addition, our primary goal was to assess how lifestyle behaviors differ between high and low-risk individuals after accounting for nonmodifiable or less modifiable factors. Therefore, we performed 1:1 propensity score matching (PSM) based on sociodemographic characteristics to assess the impact of lifestyle behaviors on ASCVD risk.

The propensity score was calculated with the following variables: sex, age, job or marital status, residential area, and household income. In descriptive analyses of two-group comparisons, continuous variables were presented as median and IQR, and categorical variables were presented as proportions (%). Differences between high and low ASCVD groups were compared using the Mann-Whitney *U* test for continuous variables after a normality test, and the chi-square test for categorical variables.

We used 5 ML algorithms for the prediction of high ASCVD risk: logistic regression (LR), support vector machine, random forest (RF), extreme gradient boosting (XGB), and light gradient boosting (LGB) model. We specifically included ensemble methods, particularly RF, XGB, and LGB models. These ensemble ML models can effectively capture complex, nonlinear relationships and high-order interactions between various risk factors (eg, lifestyle, demographic, and clinical variables) without requiring explicit prespecification [22]. In addition, they include built-in regularization techniques (like L1 and L2 penalties) to mitigate overfitting, a common concern with complex medical data [23]. In the ML model, categorical variables (including sex [after PSM, though balanced], job category, marital status, residential area, alcohol consumption frequency category, smoking status, and weight change category) were retained in their original numeric coding format. Continuous variables (including age [after PSM], omega-3 intake, sodium intake, BMI, LDL cholesterol, and time spent sitting) were scaled to a range of [0, 1] using min-max normalization. In this study, we adopted a strict data separation strategy for model development and evaluation. First, the entire dataset was randomly partitioned into a training set (80%) and a test set (20%), and stratification was applied to the partitioning to ensure that both sets retained the same class distribution as the original data. We also fixed the random seed to ensure the reproducibility of the data. Then, we performed a 5-fold stratified cross-validation on the training dataset only. In this process, we divided the training data into five equal-sized folds and repeated it five times, using 4-fold for training and one for validation in each iteration. Based on the results of this cross-validation, the optimal model structure and hyperparameters with the lowest loss were selected. Hyperparameter optimization for each of the five ML models (LR, support vector machine, RF, XGB, and LGB) was performed using Optuna, an automated hyperparameter optimization framework [24]. We used Optuna’s Tree-structured Parzen Estimator sampler, a Bayesian optimization approach, to efficiently search for optimal hyperparameter combinations. For each model, optimization was conducted over 100 trials, aiming to minimize the loss obtained from the 5-fold cross-validation within the training dataset. The specific hyperparameters tuned for each model, their respective search spaces (eg, ranges for continuous parameters and choices for categorical parameters), and the final optimal configurations identified by Optuna and used for training are all detailed in Table S1 in Multimedia Appendix 1. We evaluated the final model using a separate test set (20% of the total data). This is to objectively measure the generalization performance of the model. To calculate CIs for the ML model’s performance metrics, we used the bootstrapping method. The 95% CIs were calculated by repeating the bootstrapping samples 1000 times with 50% of the data as restoration extraction from the test dataset. Understanding the performance of ML models is becoming increasingly important, which highlights the growing significance of explainable artificial intelligence in interpreting model results. We used the SHapley Additive exPlanations (SHAP) value to visualize the importance and relationships among the input features of the model. In addition to the area under the receiver operating characteristics curve (AUROC) score, accuracy, precision, recall, *F*_1_-score, specificity, and area under the precision-recall curve were calculated to assess the performance of the developed ML models.

We conducted a multivariable LR analysis as a sensitivity test to verify whether the ML model’s findings on high ASCVD risk were consistent with conventional statistical methods. Univariable LR analysis was first performed on 1:1 matched patients (high vs low ASCVD risk) to identify lifestyle and sociodemographic factors associated with high ASCVD risk. Variables with a *P* value <.05 in the univariable model were entered into the multivariable model, and their significance was reported as adjusted odds ratios with 95% CIs.

## Results

### Baseline Characteristics

**Figure 1.**
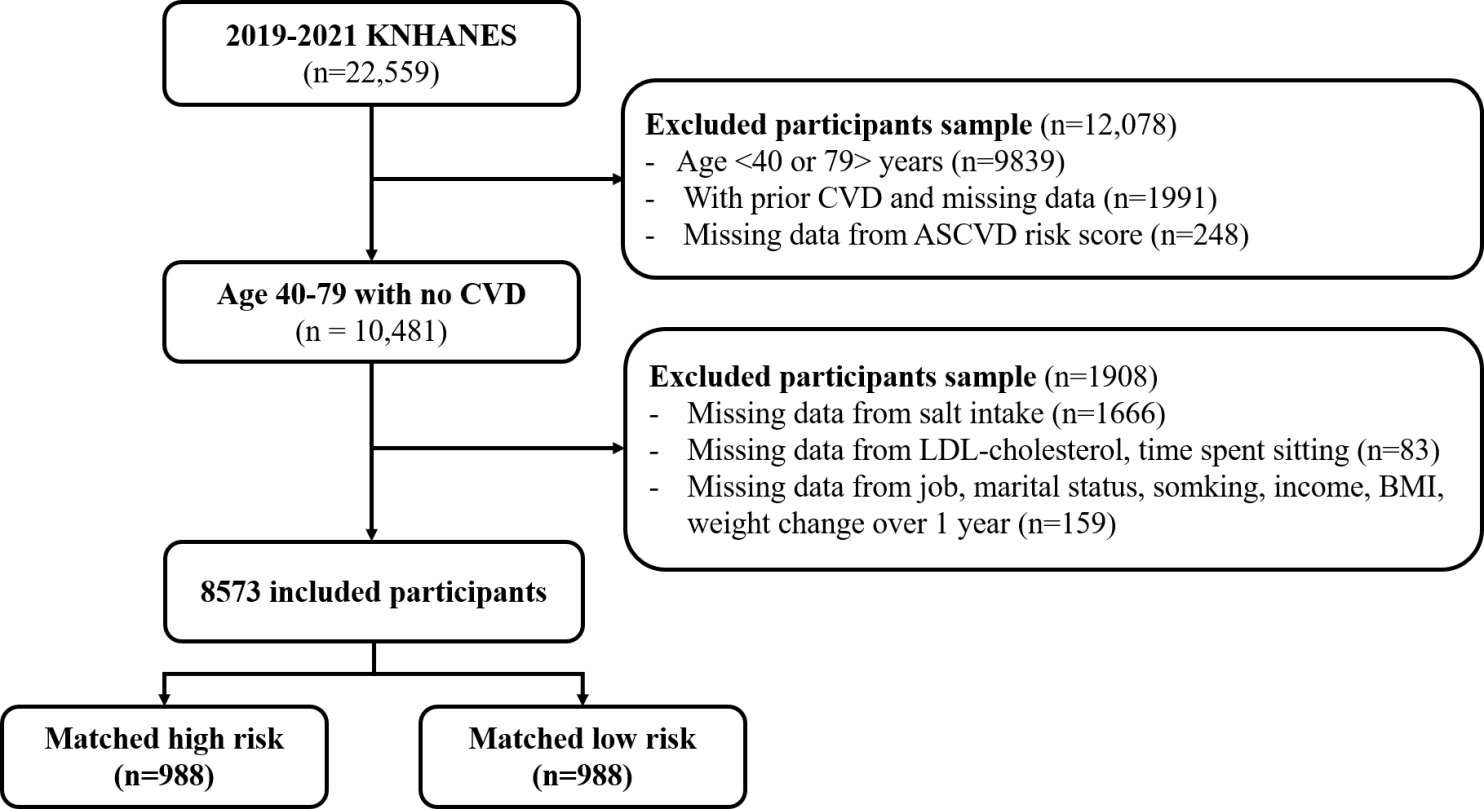
Flowchart of the participants. ASCVD: atherosclerotic cardiovascular disease; CVD: cardiovascular disease; KNHANES: Korea National Health and Nutrition Examination Survey; LDL: low-density lipoprotein.

Of the 8573 participants, the proportion of the high ASCVD risk group was 41.7% (3578/8573), and 5059 (59%) participants were women (Table 1). As detailed in Table 1, prior to PSM, substantial baseline differences were evident across nearly all measured characteristics. Notably, the high ASCVD risk group was significantly older (median age 68.0, IQR 62.0-73.0 vs 51.0, IQR 45.0-58.0 y; *P*<.001) and comprised a much higher proportion of men (61.9% vs 26%; *P*<.001) compared to the low-risk group. Significant disparities also existed in socioeconomic factors (job distribution, marital status, residential area, and income) and various lifestyle behaviors, including higher rates of frequent alcohol consumption and smoking, lower omega-3 intake, higher BMI, lower physical activity levels, and differences in weight change patterns and sitting time (all *P*≤.02). The propensity score distribution and standardized differences before and after PSM for the variables used in propensity score calculation are presented in Figures S1 and S2 in Multimedia Appendix 1. Using the 1:1 PSM method, 1976 patients were included in the final population for analysis, ensuring no significant differences in sociodemographic variables between the two groups, except for lifestyle behaviors. Following 1:1 PSM, which successfully balanced the groups for key demographic and socioeconomic confounders (sex, age, job, marital status, residential area, and income; all *P*>.20), several crucial differences in lifestyle behaviors remained statistically significant. Specifically, even after matching, the high ASCVD risk group demonstrated markedly higher rates of frequent alcohol consumption (25.3% vs 18.3 %; *P*<.001) and current smoking (42.2% vs 6.3%; *P*<.001). Furthermore, this group had significantly lower daily omega-3 intake (median 1.3, IQR 0.7-2.3 vs 1.5, IQR 0.9-2.6 g; *P*<.001) and a higher BMI (median 25.1, IQR 23.2-27.3 vs 23.9, IQR 21.9-25.8 kg/m²; *P*<.001). They also reported engaging in less physical activity (16.6% active vs 22% active; *P*=.003). Interestingly, the high-risk group reported slightly less time spent sitting compared to the low-risk group after matching (median 8, IQR 5-10 h per day; *P*=.003). Notably, the initial significant differences observed in sodium intake and LDL cholesterol levels before matching were no longer significant after PSM (*P*=.15 and *P*=.12, respectively), suggesting these factors were partly confounded by the demographic variables adjusted for in the matching process. The flowchart of participants is presented in Figure 1.

**Table 1.** Comparison of baseline characteristics of high and low ASCVD^a^ risk groups before and after propensity score matching.

Variable	Before PSM^b^			After PSM		
	Low ASCVD risk group (n=4995)	High ASCVD group (n=3578)	*P* value	High ASCVD group (n=988)	Low ASCVD group (n=988)	*P* value
Gender (women), n (%)	3695 (74)	1364 (38.1)	<.001	493 (49.9)	482 (48.8)	.65
Age (years), median (IQR)	51.0 (45.0‐58.0)	68.0 (62.0‐73.0)	<.001	59.0 (54.0‐65.0)	61.0 (52.0‐67.0)	.20
Job, n (%)			<.001			.62
Nonmanual workers	1886 (37.8)	1543 (43.1)		435 (44)	454 (46)	
Other workers	1632 (32.7)	1641 (45.9)		355 (35.9)	350 (35.4)	
Manual workers	1477 (29.6)	394 (11)		198 (20)	184 (18.6)	
Marital status (without spouse), n (%)	867 (17.4)	922 (25.8)	<.001	229 (23.2)	235 (23.8)	.36
Residential area (urban), n (%)	881 (17.6)	1001 (28)	<.001	216 (21.9)	234 (23.7)	.93
Income (10,000 won per month), median (IQR)	492 (292‐700)	230 (109-450)	<.001	360 (200‐614)	355 (193‐600)	.98
Alcohol drinking (≥2 per week), n (%)	839 (16.8)	873 (24.4)	<.001	181 (18.3)	250 (25.3)	<.001
Smoking, n (%)	442 (8.8)	915 (25.6)	<.001	62 (6.3)	417 (42.2)	<.001
Omega-3 intake (g per day), median (IQR)	1.4 (0.8‐2.3)	1.3 (0.7‐2.3)	<.001	1.5 (0.9‐2.6)	1.3 (0.7‐2.3)	<.001
Sodium intake (g per day), median (IQR)	2.8 (1.9‐4.0)	2.9 (1.9‐4.2)	.02	3.1 (2.0‐4.2)	2.9 (2.0‐4.2)	.15
BMI (kg/m²), median (IQR)	23.5 (21.4‐25.9)	24.4 (22.5‐26.5)	<.001	23.9 (21.9‐25.8)	25.1 (23.2‐27.3)	<.001
Weight change over 1 year, n (%)			<.001			.14
Weight loss	528 (10.6)	506 (14.1)		116 (11.7)	121 (12.2)	
No weight change	3171 (63.5)	2584 (72.2)		703 (71.2)	666 (67.4)	
Weight gain	1296 (25.9)	488 (13.6)		169 (17.1)	201 (20.3)	
LDL^c^ (mg/dL), median (IQR)	120 (97‐143)	110 (85‐135)	<.001	114 (91‐139)	116 (92‐143)	.12
Physical activity, n (%)	1074 (21.5)	534 (14.9)	<.001	217 (22)	164 (16.6)	.003
Time spent sitting (hours/day), median (IQR)	8.0 (5.0‐10.0)	8.0 (5.8‐11.0)	<.001	8.0 (5.0‐10.0)	8.0 (5.7‐10.9)	.003

a ASCVD: atherosclerotic cardiovascular disease.

b PSM: propensity score matching.

c LDL: low-density lipoprotein.

### Evaluation Outcomes: Performance of ML Model for ASCVD Risk

**Figure 2.**
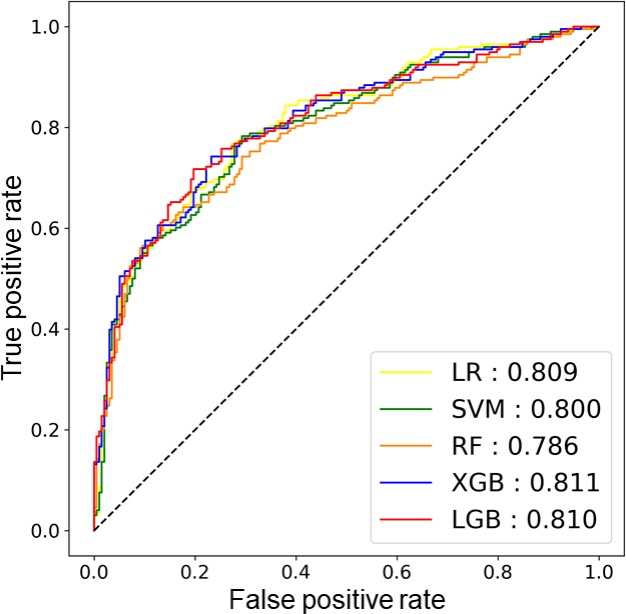
The area under the receiver operating characteristics curve values for 5 machine learning models for predicting high atherosclerotic cardiovascular disease risk (≥7.5% of 10-year risk) in the test dataset. LGB: light gradient boosting; LR: logistic regression; RF: random forest; SVM: support vector machine; XGB: extreme gradient boosting.

The 1976 matched patients were allocated to the training and test datasets in an 8:2 ratio while maintaining the same ASCVD risk proportion. A comparison of variables between these two groups is presented in Table S2 in Multimedia Appendix 1, showing no significant differences in variable distribution. Figure 2 shows the result of ML performance for predicting high ASCVD risk in the test dataset, with XGB showing the highest AUROC (0.811, 95% CI, 0.748‐0.867). Table 2 presents the other performances of the five ML models; the LGB model demonstrated the best performance, including accuracy, recall, and *F*_1_-score. The high AUROC values achieved, particularly by XGB (0.811) and LGB (0.810), indicate valid discriminative power, suggesting these models are capable of effectively distinguishing between individuals at high versus low 10-year ASCVD risk based on the included lifestyle and clinical factors. Additionally, the LGB model achieved the highest recall (0.692), meaning it correctly identified approximately 69% of the high-risk individuals in the test set. The superior *F*_1_-score (LGB 0.735) and area under the precision-recall curve score (LGB 0.829, XGB 0.828) in the test dataset showed the models’ robust performance, even in a real-world imbalanced dataset.

**Table 2.** Overall performance of the 5 machine learning models for high ASCVD^a^ risk prediction on the test dataset with propensity score matching^b^.

Model	LR^c^ (95% CI)	SVM^d^ (95% CI)	RF^e^ (95% CI)	XGB^f^ (95% CI)	LGB^g^ (95% CI)
Accuracy	0.730 (0.672‐0.788)	0.730 (0.672‐0.788)	0.732 (0.672‐0.793)	0.722 (0.662‐0.783)	0.750 (0.692‐0.808)
Precision	0.776 (0.701‐0.853)	0.827 (0.750‐0.901)	0.791 (0.714‐0.870)	0.768 (0.693‐0.846)	0.783 (0.711‐0.855)
Recall	0.646 (0.556‐0.737)	0.581 (0.485‐0.677)	0.631 (0.535‐0.727)	0.636 (0.535‐0.727)	0.692 (0.596‐0.778)
*F*_1_-score	0.705 (0.630‐0.776)	0.682 (0.600‐0.757)	0.702 (0.624‐0.774)	0.696 (0.618‐0.767)	0.735 (0.663‐0.802)
AUROC^h^	0.809 (0.748‐0.867)	0.800 (0.737‐0.859)	0.786 (0.720‐0.847)	0.811 (0.748‐0.867)	0.810 (0.749‐0.869)
Specificity	0.813 (0.737‐0.889)	0.879 (0.808‐0.939)	0.833 (0.758‐0.909)	0.808 (0.727‐0.879)	0.808 (0.727‐0.879)
AUPRC^i^	0.811 (0.741‐0.881)	0.803 (0.730‐0.873)	0.805 (0.737‐0.868)	0.828 (0.766‐0.884)	0.829 (0.771‐0.886)

a ASCVD: atherosclerotic cardiovascular disease.

b Data are represented as averaged value with 95% CI.

c LR: logistic regression.

d SVM: support vector machine.

e RF: random forest.

f XGB: extreme gradient boosting.

g LGB: light gradient boosting.

h AUROC: area under the receiver operating characteristics curve.

i AUPRC: area under the precision-recall curve.

### Variable Importance in ML Model

**Figure 3.**
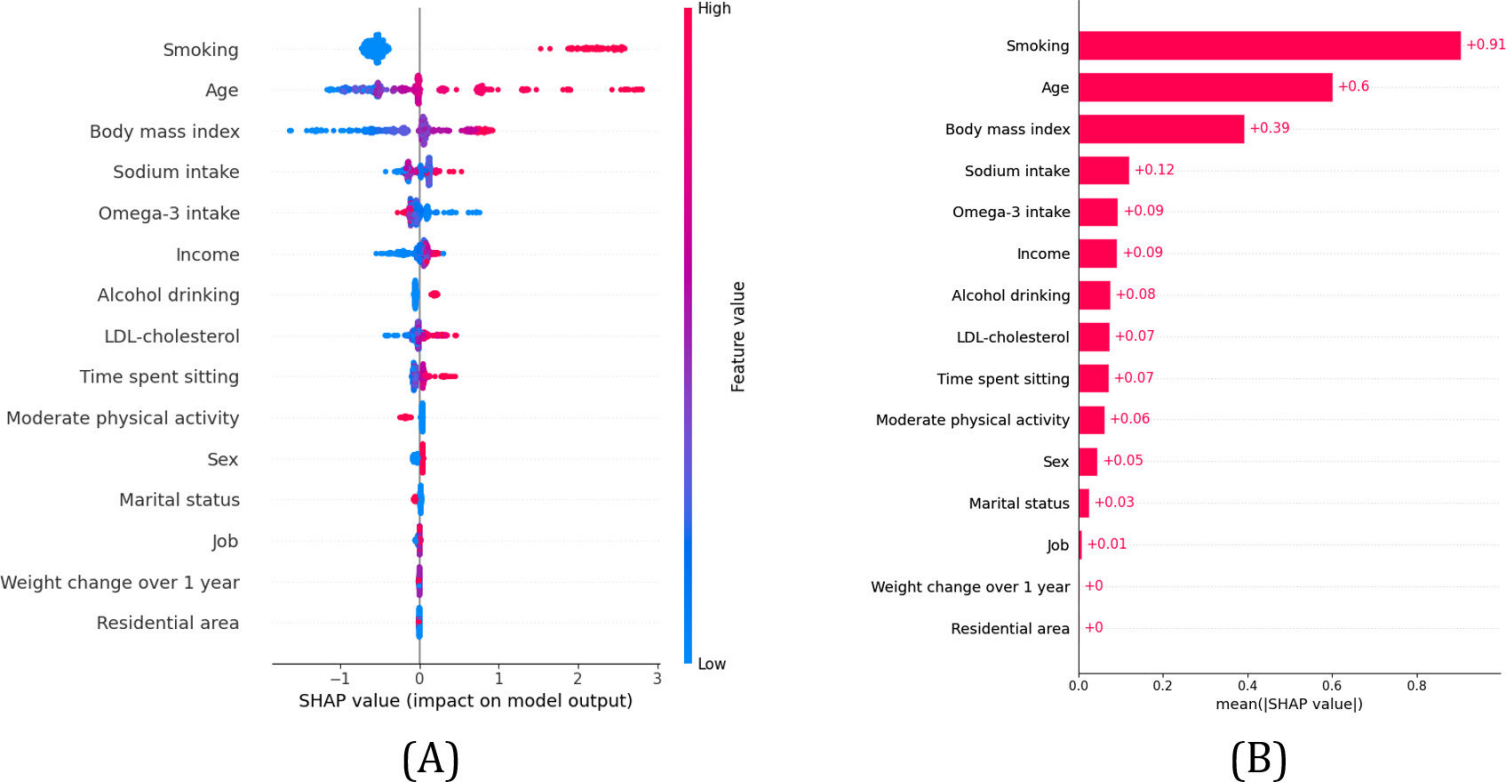
Variable importances of extreme gradient boosting models for high ASCVD risk in the test dataset. (A) Importance matrix plot showing the direction of the relationship between an input variable and high ASCVD risk. (B) Shapley Additive exPlanations summary plot of lifestyle variables predictive features of the ML model. As demonstrated by the color bar, higher values are shown in red, while lower values are shown in blue. ASCVD: atherosclerotic cardiovascular disease; LDL: low-density lipoprotein; ML: machine learning.

To visualize the importance of variables included in the ML model, we presented SHAP values for the XGB model used to predict high ASCVD risk (Figure 3). Current smoking and age were the strongest predictors of high ASCVD risk over 10 years. Among lifestyle factors, BMI, sodium or omega-3 intake, and LDL cholesterol were also associated with increased risk. In contrast, factors such as “time spent sitting,” physical activity, sex, and marital status had a relatively smaller impact. The SHAP values for the LGB model, presented in Figure S3 in Multimedia Appendix 1, show a similar pattern of variable importance as the XGB model.

### Associations of Lifestyle Risk Factors With 10-Year ASCVD Risk

For sensitivity analysis, we examined whether the ML model’s results were consistent with binary LR (Table 3) using high ASCVD risk as the outcome. Multivariable binary LR showed that smoking and BMI were positively associated with high ASCVD risk, while omega-3 intake and physical activity were significantly negatively associated. Compared to the SHAP values of the XGB and LGB models, factors such as smoking status, BMI, and omega-3 intake showed similar associations in the multivariable binary LR model.

**Table 3.** Result of binary logistic regression analysis of lifestyle variables for predicting 10-year high ASCVD^a^ risk in propensity score matched cohort.

Variable	Univariable analysis		Multivariable analysis	
	OR^b^ (95% CI)	*P* value	Adjusted OR (95% CI)	*P* value
Gender (women)	0.96 (0.80‐1.14)	.62	—^c^	—
Age (years)	1.01 (1.00‐1.02)	.20	—	—
Job				
Manual workers	1.00 (reference)	—	—	—
Nonmanual workers	1.12 (0.88‐1.43)	.34	—	—
Other workers	1.06 (0.83‐1.36)	.64	—	—
Marital status (without spouse)	1.03 (0.84‐1.27)	.75	—	—
Residential area (urban)	1.11 (0.90‐1.37)	.33	—	—
Income (10,000 won per month)	1.00 (1.00‐1.00)	.98	—	—
Alcohol drinking (≥2 per week)	1.51 (1.22‐1.87)	<.001	0.89 (0.68‐1.15)	.37
Smoking	10.91 (8.19‐14.53)	<.001	11.52 (8.20‐15.60)	<.001
Omega-3 intake (g per day)	0.93 (0.89‐0.97)	.003	0.93 (0.88‐0.98)	.01
Sodium intake (g per day)	1.00 (1.00‐1.00)	.97	—	—
BMI (kg/m^2^)	1.13 (1.10‐1.17)	<.001	1.13 (1.09‐1.16)	<.001
Weight change over 1 year				
Weight loss	1.00 (reference)	—	—	—
No weight change	0.91 (0.69‐1.20)	.49	—	—
Weight gain	1.14 (0.82‐1.58)	.43	—	—
LDL^d^ (mg/dL)	1.00 (1.00‐1.01)	.04	1.00 (1.00‐1.00)	.67
Physical activity	0.71 (0.56‐0.89)	.003	0.61 (0.47‐0.79)	<.001
Time spent sitting (hours per day)	1.00 (1.00‐1.00)	.003	1.00 (1.00‐1.00)	.42

a ASCVD: atherosclerotic cardiovascular disease.

b OR: odds ratio.

c Not applicable.

d LDL: low-density lipoprotein.

## Discussion

### Principal Findings

This study assessed the performance of ML models, including XGB and LGB, in predicting 10-year high ASCVD risk in adults and identifying key risk factors. After adjusting for confounders using PSM, both models demonstrated high AUROC values, with LGB outperforming in accuracy, recall, and *F*_1_-score. Variable importance analysis identified smoking and age as the strongest predictors, while BMI, sodium or omega-3 intake, and LDL cholesterol also had significant variables. Multivariable LR also confirmed these findings, showing that smoking, BMI, and LDL cholesterol increased ASCVD risk, whereas omega-3 intake and physical activity were associated with lower risk.

Age is associated with increased oxidative stress, which leads to an increased susceptibility to CVD onset [25]. Excessive generation of reactive oxygen species leads to a state of oxidative stress, which is a major risk factor for the development and progression of atherosclerosis [26]. Aging is an unmodifiable ASCVD risk factor, but it can be prevented if other factors can be corrected. Cigarette smoking is widely accepted as a major risk factor for the development of clinical CVD, resulting from direct effects on atherosclerosis. Epidemiologic studies strongly support that cigarette smoking in both men and women increases the incidence of myocardial infarction and fatal coronary artery disease [27,28].

Cigarette smoking is widely accepted as a major risk factor for the development of clinical CVD, resulting from direct effects on atherosclerosis. Epidemiologic studies strongly support that cigarette smoking in both men and women increases the incidence of myocardial infarction and fatal coronary artery disease [29]. Because the chemical constituents of smoke have high oxidant and inflammatory capacities, they can directly induce endothelial damage and potentiate an inflammatory response [30]. Smoke is able to increase LDL levels through metabolic alterations and the induction of LDL oxidation due to the direct oxidant capacity of smoke components [30,31]. Obesity has been linked to persistent inflammation and oxidative stress. Oxidative stress plays a crucial role in disorders related to obesity, such as dyslipidemia and hypertension, causing CVDs [32]. The prevalence of overweight and obesity has been strongly increasing over the last few decades, and it is considered to be one of the largest challenges for public health work worldwide [33].

Interestingly, some lifestyle factors commonly associated with cardiovascular health, such as sodium intake and time spent sitting, showed weaker associations with high ASCVD risk in our final models after adjusting for confounders. For instance, while sodium intake was significantly different between groups before PSM, this difference did not persist after matching, and it did not emerge as a strong predictor in the multivariable LR or the SHAP analyses for the ML models. Similarly, “time spent sitting” did not demonstrate a strong independent association in the multivariable LR model (*P*=.42) despite being significant in the univariable analysis and showing a difference post-PSM. Furthermore, its importance ranking in the SHAP analyses was relatively low. The lack of a strong independent association between sitting time and ASCVD risk may reflect collinearity with physical activity, limitations in the variable’s definition (eg, failure to capture prolonged uninterrupted sitting), potential inaccuracies in self-reported data, and residual confounding suggested by the unexpected postmatching trend.

The American Heart Association has recently outlined a new framework that focuses on defining and optimizing cardiovascular health through the adoption of 8 simple health components: healthy diet, engaging in regular physical activity, avoidance of nicotine, healthy sleep, and healthy levels of blood lipids, glucose, and blood pressure [34]. Most clinical guidelines for the primary or secondary prevention of ASCVD emphasize the importance of lifestyle modification. These lifestyle changes are also an important part of public health policy for CVD prevention. Therefore, a lifestyle approach to ASCVD risk may be important for accurate prediction and prioritization of treatment strategies for prevention.

Beyond simply ranking variable importance, the application of SHAP analysis provides crucial granular insights into how individual factors contribute to the predicted ASCVD risk for each patient, offering significant advantages over traditional “black-box” models. Our SHAP results (Figure 3 and Figure S3 in Multimedia Appendix 1) confirm that current smoking and age are not just important, but consistently exert the strongest positive influence toward a high-risk prediction across the cohort. The magnitude of the positive SHAP values associated with current smoking underscores its overwhelming impact, reinforcing the paramount importance of smoking cessation as a primary prevention strategy from a clinical standpoint. Similarly, the consistent positive contribution of increasing age highlights the nonmodifiable baseline upon which lifestyle interventions must act. Furthermore, the SHAP visualizations offer nuances beyond simple correlation. Especially, higher omega-3 intake consistently generated negative SHAP values, indicating its protective effect by lowering the predicted risk score. While LDL cholesterol’s influence appeared less dominant after PSM in the LR, SHAP analysis still identified it as a contributor, potentially highlighting its relevance within the complex interplay captured by the ML model.

### Limitations

This study has several limitations. First, it is a cross-sectional study, which does not allow for establishing causal relationships. While our ML models identify strong associations and predictive patterns (eg, smoking status being highly predictive of high ASCVD risk), this design prevents us from definitively concluding that altering a specific lifestyle factor will cause a change in ASCVD risk. For instance, the model might show that higher omega-3 intake is associated with lower predicted risk, but we cannot ascertain from this data alone whether increasing omega-3 intake would directly lead to a reduction in an individual’s true risk, or if the association is partly due to other unmeasured confounding factors linked to both omega-3 intake and cardiovascular health. This limitation significantly impacts the direct translation of our ML model’s findings, particularly the feature importance results (like SHAP values), into prescriptive clinical actions. Second, some variables were measured by questionnaire, which may lead to underestimation or overestimation for the prediction of ASCVD risk. Especially in the case of nutrition, it is particularly difficult to accurately reflect daily intake. Third, the study involved a secondary analysis of data from the KNHANES, so it was not possible to include all lifestyle and socioeconomic status variables in the analysis. Finally, we excluded 1908 participants (approximately 18% of the initially eligible cohort after age screening) due to missing data on one or more key analytical variables (sodium intake, LDL-cholesterol, time spent sitting, etc). This exclusion was based on a complete case analysis approach, meaning only participants with complete records for all variables used in the models were included. Multiple imputation techniques could potentially allow for the inclusion of this population and might mitigate some of the selection bias. Future studies should investigate the impact of handling missing data via multiple imputation compared to complete case analysis, specifically assessing potential improvement in the performance metrics of the developed ML models. Nevertheless, the strength of this study is that it used representative and reliable data from KNHANES to predict modifiable lifestyle risk factors using an ML approach.

### Conclusions

This study demonstrates that ML models are effective tools for assessing ASCVD risk and highlights the significant impact of lifestyle factors such as smoking, BMI, and omega-3 intake. These findings highlight the significant clinical informatics potential for integrating the developed interpretable ML models into electronic health records, clinical decision support tools, and digital health platforms to enable dynamic, personalized ASCVD risk assessment and guide targeted lifestyle interventions within routine clinical practice. Longitudinal studies are needed to establish causal links between the identified lifestyle factors and ASCVD development in a multiethnic population. Furthermore, additional measures of lifestyle with wearable devices could enhance the clinical utility of the ML model.

## Supplementary material

10.2196/74415 Multimedia Appendix 1 Supplementary figures and tables.

## Abbreviations

ASCVD: atherosclerotic cardiovascular disease
AUROC: area under the receiver operating characteristic curve
CVD: cardiovascular disease
KDCA: Korea Disease Control and Prevention Agency
KNHANES: Korea National Health and Nutrition Examination Survey
LDL: low-density lipoprotein
LGB: light gradient boosting
LR: logistic regression
ML: machine learning
PCE: pooled cohort equation
PSM: propensity score matching
RF: random forest
SHAP: SHapley Additive exPlanations
XGB: extreme gradient boosting
