# Supplementary material for: Machine Learning–Based Analysis of Lifestyle Risk Factors for Atherosclerotic Cardiovascular Disease: Retrospective Case-Control Study
Source: JMIR Med Inform. 2025 Aug 7;13:e74415. doi: 10.2196/74415 (PMC12330983; doi:10.2196/74415)
Supplement: Multimedia Appendix 1 [file medinform-v13-e74415-s001.docx]

**Figure S1.** Standardized mean difference of variables before and after propensity score matching.


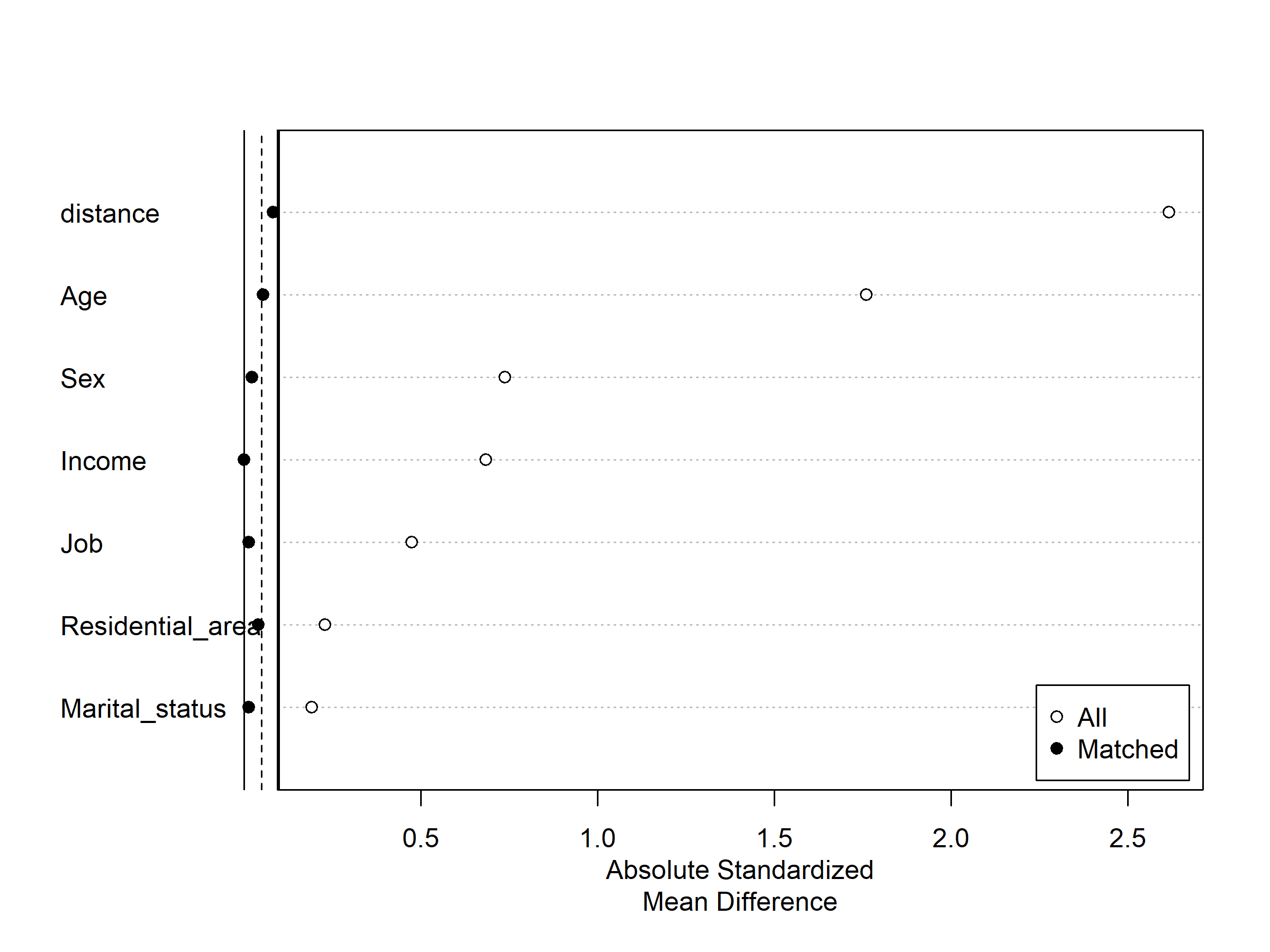


**Figure S2.** Distribution of propensity scores of the participants (before and after propensity score matching).


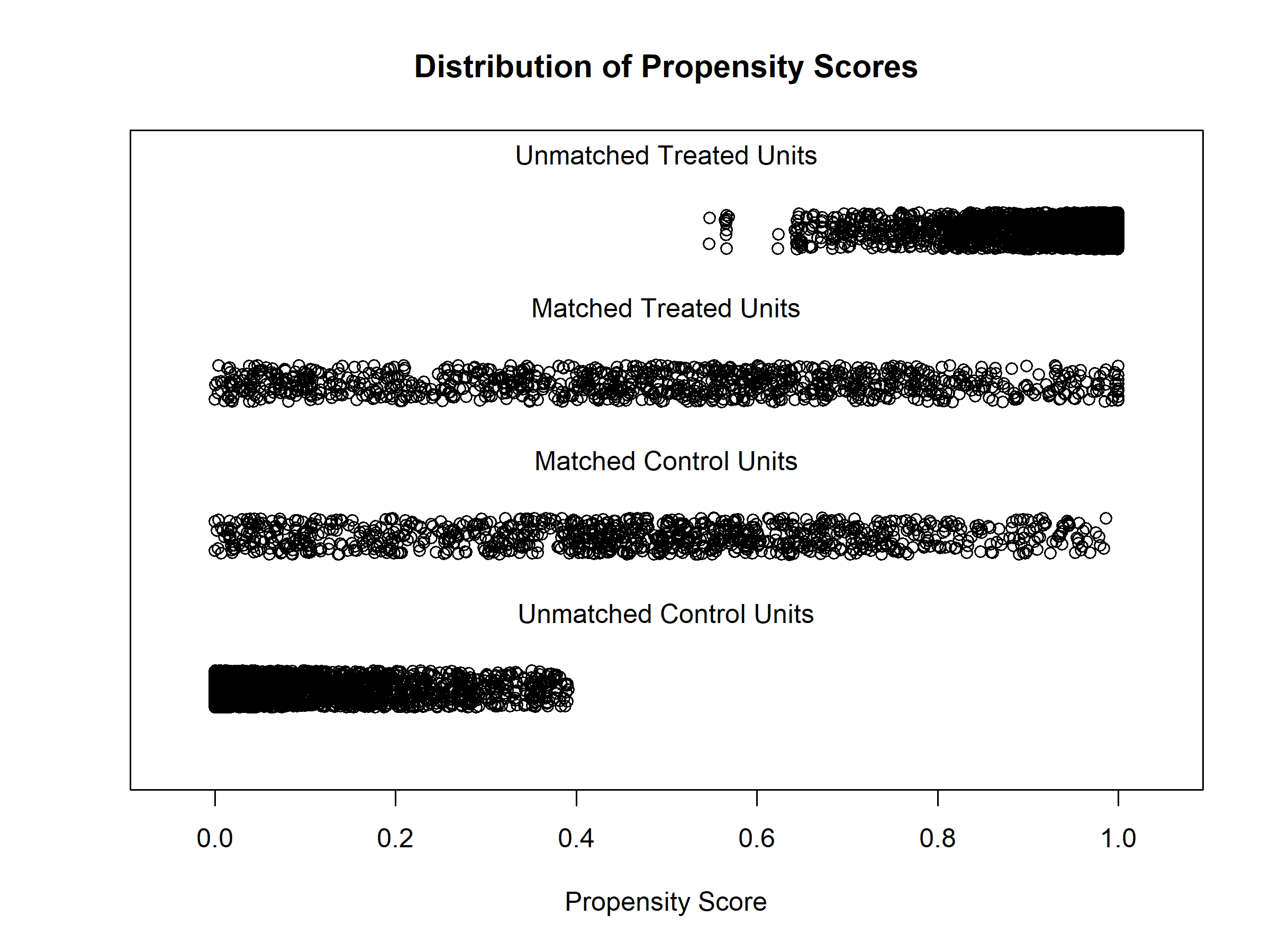


**Table S1.** Hyperparameter search spaces and final optimal values for machine learning models.

| Model | Hyperparameter | Search Space | Final optimal value |
| --- | --- | --- | --- |
|  |  |  |  |
| LR^a^ | C (inverse regularization parameter) | (0.01, 10, step=0.1) | 1.11 |
|  | class_weight | balanced | balanced |
|  | max_iter | (1, 5000) | 952 |
| SVM^b^ | C (Regularization parameter) | (0.01, 10, step=0.01) | 1.640 |
|  | class_weight | balanced | balanced |
|  | Kernel | linear | linear |
| RF^c^ | n_estimators | (10, 1000) | 46 |
|  | max_depth | (2, 20) | 17 |
|  | max_leaf_nodes | (2, 20) | 20 |
|  | min_samples_leaf | (1, 200) | 12 |
|  | class_weight | balanced | balanced |
| XGB^d^ | n_estimators | (100, 2000) | 767 |
|  | learning_rate | (0.01, 0.05) | 0.01388 |
|  | max_depth | (1, 10) | 2 |
|  | booster | gbtree | gbtree |
|  | Class_weight | balanced | balanced |
|  | gamma (min split loss) | (0, 1) | 1 |
|  | min_child_weight | (1, 20) | 1 |
|  | max_bin | (2, 512) | 255 |
| LGB^e^ | n_estimators | (100, 2000) | 1831 |
|  | learning_rate | (0.01, 0.05) | 0.04980 |
|  | max_depth | (1, 10) | 1 |
|  | Num_leaves | (31, 200) | 169 |
|  | class_weight | balanced | balanced |
|  | early_stopping_round | (50, 50) | 50 |
|  | min_child_samples | (1, 3) | 3 |
|  | min_data_in_leaf | (20, 200) | 20 |
|  | verbosity | -1 | -1 |
|  | Subsample (or bagging fraction) | (0.5, 1.0) | 0.56084 |
|  | Colsample_bytree (or feature_fraction) | (0.1, 1.0) | 0.98470 |
|  | reg_alpha (L1 regularization) | (1e-8, 1.0) | 1.58458e-06 |
|  | reg_lambda (L2 regularization) | (1e-8, 1.0) | 3.52007e-08 |

^a^LR, logistic regression.

^b^SVM: support vector machine.

^c^RF: random forest.

^d^XGB: extreme gradient boosting.

^e^LGB: light gradient boosting.

**Table S2.** Comparison of baseline characteristics of the training and test dataset after propensity score matching.

| Variable | Training dataset (N=1580) | Test dataset (N=396) | *P* |
| --- | --- | --- | --- |
|  |  |  |  |
| Sex, women | 770 (48.7%) | 205 (51.8%) | 0.306 |
| Age, median (IQR^a^) | 59.0 (53.0–66.0) | 61.0 (54.0–65.0) | 0.074 |
| Job |  |  | 0.321 |
| Non-manual workers | 704 (44.6%) | 185(46.7%) |  |
| Other workers | 560 (35.4%) | 145 (36.6%) |  |
| Manual workers | 316 (20.0%) | 66 (16.7%) |  |
| Marital status, without spouse | 372 (23.5%) | 92 (23.2%) | 0.948 |
| Residential area, urban | 1225 (77.5%) | 301 (76.0%) | 0.563 |
| Income, 10,000 won/month | 368 (200–607) | 333 (192–601) | 0.437 |
| Alcohol drinking, ≥2/week | 360 (22.8%) | 71 (17.9%) | 0.043 |
| Smoking | 397 (25.1%) | 82 (20.7%) | 0.077 |
| Omega-3 intake, g/day | 1.4 (0.8–2.3) | 1.5 (0.9–2.5) | 0.148 |
| Sodium intake, g/day | 3.0 (2.0–4.2) | 2.9 (2.0–4.3) | 0.536 |
| Body mass index, kg/m² | 24.4 (22.5–26.7) | 24.6 (22.6–26.2) | 0.671 |
| Weight change over 1 year |  |  | 0.846 |
| Weight loss | 187 (11.8%) | 50 (12.6%) |  |
| No weight change | 1094 (69.2%) | 275 (69.4%) |  |
| Weight gain | 299 (18.9%) | 71 (17.9%) |  |
| LDL^b^, mg/dL | 115 (92–140) | 117 (92–142) | 0.650 |
| physical activity | 302 (19.1%) | 79 (19.9%) | 0.760 |
| Time spent sitting, hour/day | 8.0 (5.0–10.0) | 8.0 (5.0–10.0) | 0.890 |
| High ASCVD risk | 790 (50.0%) | 198 (50.0%) | 1.000 |

Data are represented as median (interquartile change) or number (percent) as appropriate.

^a^IQR: interquartile change.

^b^LDL: low-density lipoprotein.

**Figure S3.** Variable importances of light gradient boosting models for high atherosclerotic cardiovascular disease risk in the test dataset.


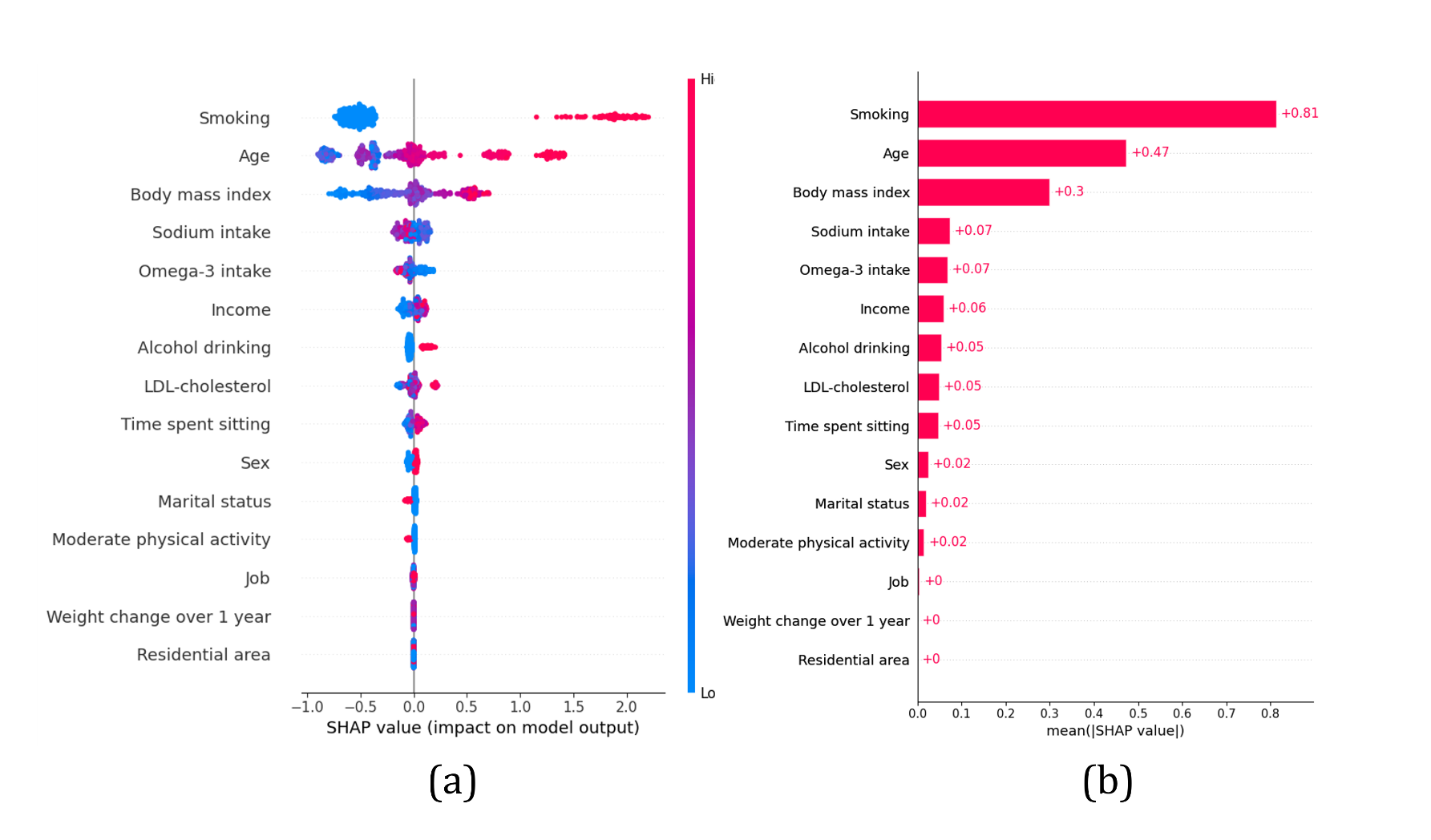


(a) Importance matrix plot showing the direction of the relationship between a input variable and high atherosclerotic cardiovascular disease risk (b) Shapley Additive exPlanations (SHAP) summary plot of lifestyle variables predictive features of the ML model. As demonstrated by the colorbar, higher values are shown in red, while lower values are shown in blue.
